# Geriatric co-managed prehabilitation before esophagectomy in patients aged ≥ 65 years with esophageal cancer: a prospective feasibility study with matched historical controls

**DOI:** 10.1007/s00423-026-04285-y

**Published:** 2026-09-29

**Authors:** Linus Alexander Völker, Emily Haak, Hans Schlößer, Christiane J. Bruns, Pauline Schumacher, Wolfgang Schröder, Paul Thomas Brinkkoetter

**Affiliations:** 1 Department II of Internal Medicine, Center for Molecular Medicine Cologne, Faculty of Medicine, University Hospital Cologne, University of Cologne, Cologne, Germany; 2 Cologne Cluster of Excellence on Cellular Stress Responses in Ageing-Associated Diseases, Cologne, Germany; 3 Center of Excellence in Nephrology, University Hospitals Aachen, Cologne, Dusseldorf, Aachen, Cologne, Dusseldorf, North Rhine-Westphalia Germany; 4 Department of Internal Medicine and Geriatrics, Evangelisches Klinikum Köln Weyertal, Cologne, Germany; 5 Department of General, Visceral, Cancer and Transplant Surgery, University Hospital of Cologne, Cologne, Germany; 6 Department of General, Visceral and Cancer Surgery, Helios University Hospital Wuppertal, Wuppertal, Germany

**Keywords:** Prehabilitation, Esophagectomy, Esophageal neoplasms, Geriatric assessment, Feasibility studies, Perioperative care

## Abstract

**Purpose:**

Older patients undergoing esophagectomy remain at high risk for perioperative morbidity. This study evaluated the feasibility, safety and adherence of a multimodal prehabilitation program integrated with geriatric co-management in patients aged ≥ 65 years scheduled for transthoracic esophagectomy and explored perioperative outcomes.

**Methods:**

In this single-center prospective feasibility study, patients aged ≥ 65 years scheduled for transthoracic esophagectomy completed a 4-week home-based program of endurance, respiratory, resistance and balance training combined with comprehensive geriatric assessment and medication optimization. Feasibility outcomes were retention, adherence and data completeness. Perioperative outcomes were compared with a 2:1 matched historical control cohort using cluster-robust linear regression for continuous outcomes and Firth penalized logistic regression for binary outcomes.

**Results:**

Twenty-six patients were enrolled; 22 completed the intervention and were matched to 42 control records. At the first check-in 61.5% reported complete and 84.6% complete or mostly complete adherence; documentation was missing for 69.2% at the second. Despite matching, controls had more favorable performance status (*p* = 0.023) and comorbidity burden (*p* = 0.014). Postoperative complications, anastomotic leakage, hospital mortality and length of stay did not differ significantly. Intensive care unit (ICU) readmission was lower after prehabilitation (4.5% vs. 26.2%; odds ratio 0.19, 95% confidence interval 0.02–0.90; *p* = 0.035), but not after adjustment for surgical approach (adjusted odds ratio 0.30; *p* = 0.172).

**Conclusion:**

Multimodal prehabilitation with geriatric co-management was feasible and safe in this population. Overall postoperative morbidity was unchanged. The lower ICU readmission rate did not withstand adjustment and is hypothesis-generating requiring an adequately powered randomized trial. Future programs need more robust adherence monitoring.

**Trial registration:**

ClinicalTrials.gov NCT05167682, registered 9 December 2021, retrospectively registered.

**Supplementary Information:**

The online version contains supplementary material available at https://doi.org/10.1007/s00423-026-04285-y.

## Introduction

Despite advances in surgical techniques and postoperative management, transthoracic esophagectomy remains associated with substantial postoperative morbidity. Contemporary international registry data report postoperative complications in approximately 53–75% of patients, whereas major complications, defined as Clavien–Dindo grade ≥IIIb, occur in roughly 16–19%; pulmonary complications including pneumonia and anastomotic leakage remain frequent, with reported rates of approximately 11–38% and 7–26%, respectively, after Ivor Lewis esophagectomy [1, 2]. The postoperative complication rate not only correlates with the number and severity of individual comorbidities but also with the age of the patient [3]. Even though numerical age is not in itself a contraindication for esophagectomy, preoperative risk assessment becomes particularly important with increasing age [4]. In addition to the comorbidities often associated with age, the underlying disease of esophageal cancer also presents specific risk factors such as malnutrition [5].

Beyond achieving complete tumor resection, a primary goal of transthoracic esophagectomy is to minimize postoperative complications. This is essential for ensuring that patients with locally advanced tumors can initiate adjuvant therapy as early as possible. In this context, the concept of prehabilitation emerges as a potential strategy to mitigate postoperative morbidity [6–8]. Prehabilitation aims to enhance surgical readiness and shorten recovery times through preemptive screening for frailty and a targeted preoperative intervention. Multimodal prehabilitation commonly targets several modifiable risk domains, including physical endurance and strength, respiratory capacity, nutritional status, psychological resilience, and medication-related risks such as polypharmacy. Current guideline recommendations remain cautious, as available studies differ substantially in patient populations, intervention components, duration, supervision, and outcome definitions. Consequently, no universally accepted standardized prehabilitation protocol has been established for this surgical population [9]. Lack of standardization and variable adherence to home-based prehabilitation programs are key reasons why evidence for their effect on postoperative morbidity remains limited [10, 11].

We therefore conducted a prospective single-center feasibility study to assess whether a structured, home-based, multimodal prehabilitation program integrated with geriatric co-management can be implemented in patients aged 65 years and older before transthoracic esophagectomy. In addition, exploratory perioperative outcomes were compared with a matched historical control cohort.

## Materials and methods

### Study design and setting

This was a single-center, prospective feasibility study conducted at the Department of General, Visceral, Thoracic and Transplantation Surgery and the Department of Internal Medicine II and Ageing Medicine, University Hospital Cologne, Germany. The study evaluated the effects of a structured, multimodal prehabilitation program in patients aged ≥ 65 years scheduled for elective esophagectomy. Outcomes from the intervention cohort were compared to a historical matched-control group in a 2:1 ratio. The study was approved by the local ethics committee prior to patient recruitment, and all participants provided written informed consent (see Statements and Declarations). The study was retrospectively registered at ClinicalTrials.gov (NCT05167682) on 9 December 2021.

### Participants

Eligible patients were adults 65 years of age and older scheduled for elective esophagectomy for esophageal carcinoma, with a minimum of four weeks available prior to the planned surgical date. The prehabilitation program was intended to be performed after neoadjuvant therapy prior to esophagectomy. Exclusion criteria included severe physical or psychological limitations precluding participation in the exercise program, and any medical contraindication for physical training as determined by the treating surgical and geriatric teams.

### Intervention: prehabilitation program

The multimodal prehabilitation was co-supervised by the treating surgeon and a board-certified geriatrician. The program included four core components: (1) aerobic endurance training, (2) respiratory exercises, (3) resistance (strength-endurance) training, and (4) balance and coordination exercises. The program was individually adapted according to baseline physical assessment performed by a physiotherapist, in consultation with the surgeon and geriatrician. The geriatrician performed a chart review to (1) identify potentially inadequate medications for the elderly, (2) optimize therapy of chronic disease, and (3) reduce polypharmacy. Adherence was monitored and training intensity was adjusted according to patient well-being and capability through phone calls to the patient every two weeks.

#### Aerobic endurance training

Patients were prescribed a progressive endurance exercise regimen consisting of walking, jogging intervals, or cycling, aiming for daily activity at moderate intensity. Outdoor activity was encouraged to facilitate oxygenation and general well-being. Endurance protocols were structured week-by-week, starting from low-to-moderate volumes in week 1 and progressing in intensity and duration through week 4.

#### Respiratory training

Breathing exercises were performed three times per week on non-consecutive days. Training duration increased from 10 min per session in weeks 1–2 to 15 min per session in weeks 3–4. Exercises focused on inspiratory muscle activation and facilitation of diaphragmatic breathing.

#### Resistance (strength-endurance) training

Strength exercises targeted major muscle groups, emphasizing functional movements to improve perioperative functional reserve. Examples included squats, planks and variations, and theraband-based upper body exercises such as shoulder retraction, external rotation, and diagonal lifts (“chest opener”). Exercises were performed in sets of 10–20 repetitions or in time-based intervals following a Tabata-style format. Intensity was adapted by modifying load, range of motion, or complexity.

#### Balance and coordination training

Static and dynamic balance exercises were incorporated to improve postural control and reduce fall risk. These included single-leg stance (“stork”), hip flexor stretching combined with stability tasks, and lateral movement patterns (“Schuhplattler”) integrating lower limb coordination.

#### Weekly exercise schedule

The exercise week combined aerobic, strength, and balance sessions with dedicated breathing training days. For example, in week 1 patients completed approximately 185 min of structured exercise, progressing to about 195 min by week 4. Weekend sessions included longer walks or cycling, and interval-based jogging protocols with progressive increases in running time. Evening stretching and stabilization routines complemented the aerobic elements.

### Baseline and follow-up assessments

After screening and obtaining informed consent (V1), assessments were scheduled at baseline (V2), after completion of the prehabilitation period (V3), at hospital discharge (V4), and at a 90-day follow-up visit (V5). Assessments included demographics and clinical parameters (baseline characteristics, American Society of Anesthesiologists (ASA) physical status, Eastern Cooperative Oncology Group (ECOG) performance status, Charlson Comorbidity Index (CCI), body mass index (BMI), medical comorbidities, smoking history and pack years), functional capacity (6-minute walk test supervised at baseline, self-administered preoperatively, the Duke Activity Status Index (DASI) [12], and the Global Physical Activity Questionnaire (GPAQ) [13]), nutritional and logopedic assessment (Nutritional Risk Screening (NRS) [14], bioelectrical impedance vector analysis (BIVA; seca deutschland vertrieb gmbh & co. kg., Hamburg, Germany) reported as the phase angle in degrees (°), and Ogilvie dysphagia score [15]), respiratory function via spirometry, a comprehensive geriatric assessment (Clinical Frailty Scale (CFS) [16], timed up and go (TUG) [17], de Morton Mobility Index (DEMMI) [18], social status questionnaire [19], Mini-Mental State Examination (MMSE) [20], Geriatric Depression Scale (GDS) [21], SF-36 questionnaire [22]) and prognosis via the modified Glasgow Prognostic Score (mGPS) [23]. Postoperative outcomes were recorded using the Clavien–Dindo classification as well as hospital length of stay, intensive care unit (ICU) stay, and readmission to hospital [24]. Postoperative complications were classified according to the Esophagectomy Complications Consensus Group (ECCG) definitions [25].

### Matched-control cohort

Historical controls were identified from institutional records of patients aged ≥ 65 years who underwent esophagectomy for esophageal cancer between August 2021 and December 2023, largely corresponding to the recruitment period of the intervention cohort. If possible, controls were matched at a 2:1 ratio with replacement to interventional patients using age groups (65–69, 70–74, 75–79, 80–84, > 84 years), ASA classes (ASA 1 or 2, ASA 3 or 4), types of neoadjuvant treatment (radiochemotherapy, chemotherapy, no neoadjuvant therapy), and BMI (< 30, 30–35, > 35 kg/m²) as matching criteria. No prehabilitation program was implemented in the control group.

### Statistical analysis

#### Data management and study population

Data were analyzed using R statistical software (version 4.2.2) and the following packages: readxl version 1.4.1 for Excel file import and data extraction; dplyr version 1.1.0 and tibble version 3.1.8 for data manipulation and processing; clubSandwich version 0.6.0 for cluster-robust variance estimation and inference; lme4 version 1.1.31 and lmerTest version 3.2.1 for mixed-effects modeling; logistf version 1.26.1 for Firth’s penalized logistic regression; knitr version 1.42 and kableExtra version 1.3.4 for statistical table formatting and presentation; and ggplot2 version 3.4.0 to generate figures.

#### Primary and secondary outcomes

The primary endpoints of this feasibility study were study retention (proportion of patients completing all scheduled visits), overall data completeness (proportion of assessment items successfully documented per patient), self-reported adherence to the prescribed training program, and need for training intensity adaptation.

Secondary endpoints comprised postoperative outcomes — hospital mortality, complication rate (Clavien–Dindo classification), anastomotic leakage, ICU readmission, and postoperative hospital length of stay — as well as changes in functional capacity, nutritional status, body composition, health-related quality of life, and mobility between the baseline (V2) and post-prehabilitation (V3) assessments. Ninety-day mortality was recorded in the intervention cohort, but only in-hospital mortality was available for the historical control cohort; hospital mortality was therefore used for the between-cohort comparison. As no deaths occurred between hospital discharge and day 90, the two definitions coincided. Given the concurrent administration of neoadjuvant therapy, maintenance of preoperative functional status was considered an acceptable treatment effect. No formal power calculation was carried out due to the design as a feasibility study.

#### Study groups and matching design

Three analytical cohorts were defined (see Online Resource 1): ITT — intention-to-treat group (all enrolled patients; *n* = 26), PP — per-protocol/matched cases group (patients completing intervention and successfully matched; *n* = 22), and MC — matched controls group (control patients matched to per-protocol patients; *n* = 42 records from 38 unique individuals).

#### Descriptive statistics

Continuous variables were presented as mean ± standard deviation (SD) for normally distributed data or median with interquartile range (IQR) for non-normally distributed data or small sample sizes. Categorical variables were reported as frequencies and percentages.

#### Comparative analysis

For matched-pair analysis with replacement matching, cluster-robust statistical methods were employed using the clubSandwich package. Continuous variables were analyzed using cluster-robust linear regression (CR2 sandwich estimator) with Satterthwaite degrees of freedom correction, with clustering by patient ID. Binary categorical outcomes were analyzed using Firth’s penalized logistic regression; odds ratios (OR), 95% confidence intervals (CI), and p values were all derived from the same penalized likelihood ratio test to ensure internal consistency of point estimate, interval, and p value under the sparse-data and small-cell conditions present in this cohort. Firth penalized logistic regression does not model the matched-set correlation arising from replacement matching; binary outcome results are therefore presented as descriptive estimates and should not be interpreted as inferentially adjusted for the matching structure. A pre-specified sensitivity analysis additionally adjusted for surgical approach (open, hybrid, minimally invasive, or robotic) as a factor covariate in all outcome models to assess robustness to differences in operative technique between cohorts.

#### Repeated measures analysis

To account for the presence of missing values for repeated measures at baseline and follow-up visits, a linear mixed-effects model (LMM) was employed. All independent variables were treated as continuous predictors and were centered and scaled (Z-standardization) prior to analysis to facilitate the comparison of effect sizes and to improve model convergence.

The model employed random intercepts for each participant to account for inter-individual baseline variability. Fixed effects were estimated for all continuous covariates of interest. Parameters were estimated using the restricted maximum likelihood (REML) method. The significance of the estimates was assessed using Satterthwaite’s approximation for degrees of freedom. Because outcome variables were Z-standardized prior to analysis, all estimates are expressed in SD units, providing a standardized effect scale that approximates Cohen’s d for normally distributed outcomes and may facilitate future meta-analyses and sample size calculations.

#### Missing data and sample size

Cross-sectional analyses (baseline characteristics and postoperative outcome comparisons) were performed using complete-case analysis, with valid observation counts reported explicitly (indicated as N = n when applicable). No imputation was applied to these analyses. Repeated-measures analyses (V2 to V3 comparisons) used linear mixed-effects models, which incorporate all available observations at each time-point and implicitly assume data are missing at random (MAR). In the matched control cohort, a control patient matched to more than one intervention patient contributes one record per match; all such records were retained in every analysis and no de-duplication was applied, so denominators below 42 reflect missing values only.

#### Statistical significance and reporting

Statistical significance was defined as *p* < 0.05. P values were reported to three decimal places, with values < 0.001 reported as “<0.001”. Across baseline characteristics and outcome comparisons, more than 25 statistical tests were performed. Given the exploratory nature and limited sample size, no adjustment for multiple comparisons was applied; the expected number of false-positive findings under the global null hypothesis is therefore substantial, and all results should be treated as hypothesis-generating. Patient flow was documented according to CONSORT guidelines for non-randomized studies.

## Results

### Patient enrollment

Between June 2021 and December 2023, 74 patients with esophageal carcinoma were screened for eligibility (Online Resource 1). Twenty-six patients were enrolled, corresponding to 35.1% of screened candidates. Main reasons for non-enrollment included the implementation of a conservative treatment plan (*n* = 19), transfer to an outside medical facility due to COVID-19-related suspension of elective patient tracks (*n* = 10), an urgent indication for surgery (*n* = 7), and lack of consent (*n* = 5).

Twenty-four participants ultimately underwent esophagectomy; one patient died prior to surgery and one experienced respiratory failure during anesthesia induction and did not proceed to surgery. Twenty-two of these trial patients were successfully matched to controls.

### Baseline characteristics

The demographic characteristics of the three analytical cohorts are summarized in Table 1. The mean patient age was 71.9 years in the intention-to-treat group (ITT), 71.0 years in the per-protocol/matched cases group (PP) and 70.4 years in the matched controls group (MC). In the cohorts, 73.1% in the ITT, 72.7% in the PP and 73.8% in the MC were male. No significant differences could be seen for ASA physical status classification (*p* = 0.259 for ITT vs. MC; *p* = 0.449 for PP vs. MC) or BMI (*p* = 0.796 for ITT vs. MC; *p* = 0.818 for PP vs. MC). The MC group showed more favorable scores for ECOG performance status (*p* = 0.015 for ITT vs. MC; *p* = 0.023 for PP vs. MC), CCI (*p* = 0.047 for ITT vs. MC; *p* = 0.014 for PP vs. MC) and baseline FEV1 (2.7 ± 0.7 L vs. 2.3 ± 0.6 L in the ITT and 2.4 ± 0.6 L in the PP cohort; *p* = 0.015 for ITT vs. MC; *p* = 0.044 for PP vs. MC).

**Table 1:** Baseline characteristics of the three analytical cohorts

Characteristic	ITT (*n* = 26)	PP (*n* = 22)	MC (*n* = 42)	*p* ITT vs. MC	*p* PP vs. MC
Sex [n (%)]				0.950	0.930
Female	7 (26.9%)	6 (27.3%)	11 (26.2%)		
Male	19 (73.1%)	16 (72.7%)	31 (73.8%)		
Age (years) [mean ± SD]	71.9 ± 3.8	71.0 ± 3.2	70.4 ± 3.5	0.105	0.466
BMI (kg/m²) [mean ± SD]	25.1 ± 4.7	25.1 ± 4.5	24.8 ± 3.2	0.796	0.818
ASA physical status [n (%)]				0.259	0.449
ASA 1	2 (7.7%)	2 (9.1%)	10 (23.8%)		
ASA 2	17 (65.4%)	15 (68.2%)	22 (52.4%)		
ASA 3	7 (26.9%)	5 (22.7%)	10 (23.8%)		
ECOG performance status [n (%)]	(*N* = 23)	(*N* = 20)		0.015	0.023
ECOG 0	10 (43.5%)	9 (45.0%)	31 (73.8%)		
ECOG 1	10 (43.5%)	8 (40.0%)	9 (21.4%)		
ECOG 2	2 (8.7%)	2 (10.0%)	2 (4.8%)		
ECOG 3	1 (4.3%)	1 (5.0%)	–		
Charlson Comorbidity Index [median (IQR)]	3.0 (2.0–3.8)	3.0 (2.2–4.0)	2.0 (2.0–3.0) (*N* = 38)	0.047	0.014
Serum albumin (g/L) [mean ± SD]	41.0 ± 4.1 (*N* = 24)	41.2 ± 4.1 (*N* = 21)	40.9 ± 4.7 (*N* = 35)	0.893	0.770
FEV1 (L) [mean ± SD]	2.3 ± 0.6 (*N* = 22)	2.4 ± 0.6 (*N* = 20)	2.7 ± 0.7 (*N* = 41)	0.015	0.044
Neoadjuvant therapy [n (%)]				0.862	0.970
Yes	25 (96.2%)	21 (95.5%)	40 (95.2%)		
No	1 (3.8%)	1 (4.5%)	2 (4.8%)		
Type of neoadjuvant therapy [n (%)]				1.000	1.000
CROSS (chemoradiotherapy)	19 (73.1%)	15 (68.2%)	30 (71.4%)		
FLOT (perioperative chemotherapy)	6 (23.1%)	6 (27.3%)	10 (23.8%)		
None	1 (3.8%)	1 (4.5%)	2 (4.8%)		
Type of surgery [n (%)]				0.002	0.009
Hybrid	21 (80.8%)	19 (86.4%)	19 (45.2%)		
Totally minimally invasive	1 (3.8%)	1 (4.5%)	4 (9.5%)		
Robotic	2 (7.7%)	2 (9.1%)	11 (26.2%)		
Open	–	–	8 (19.0%)		
No surgery^a^	2 (7.7%)	–	–		
COPD [n (%)]	6 (23.1%)	5 (22.7%)	N/A	N/A	N/A
Smoking status [n (%)]			N/A	N/A	N/A
Non-smoker	3 (11.5%)	3 (13.6%)			
Current smoker	8 (30.8%)	8 (36.4%)			
Ex-smoker	15 (57.7%)	11 (50.0%)			
Pack years [mean ± SD]	36.0 ± 28.0 (*N* = 21)	37.3 ± 29.9 (*N* = 18)	N/A	N/A	N/A
Coronary artery disease [n (%)]	5 (19.2%)	4 (18.2%)	N/A	N/A	N/A
Heart failure [n (%)]	12 (46.2%)	10 (45.5%)	N/A	N/A	N/A
Arterial hypertension [n (%)]	18 (69.2%)	15 (68.2%)	N/A	N/A	N/A
Atrial fibrillation [n (%)]	2 (7.7%)	2 (9.1%)	N/A	N/A	N/A
Peripheral arterial disease [n (%)]	0 (0.0%)	0 (0.0%)	N/A	N/A	N/A
Chronic kidney disease [n (%)]	0 (0.0%)	0 (0.0%)	N/A	N/A	N/A
Diabetes mellitus type 1 [n (%)]	0 (0.0%)	0 (0.0%)	N/A	N/A	N/A
Diabetes mellitus type 2 [n (%)]	7 (26.9%)	7 (31.8%)	N/A	N/A	N/A
Hypothyroidism [n (%)]	4 (15.4%)	4 (18.2%)	N/A	N/A	N/A

^a^ One patient died prior to surgery; one experienced respiratory failure at induction of anesthesia

Values are n (%), mean ± SD or median (IQR); *N* = 26 (ITT), *N* = 22 (PP) and *N* = 42 (MC) unless indicated otherwise. Continuous variables: cluster-robust linear regression (CR2); ordinal variables (ECOG, CCI): Wilcoxon rank-sum test; categorical variables: chi-squared test with simulated p value (B = 2000). Comorbidity data (COPD through hypothyroidism) were available only for the ITT and PP cohorts. All matched control analyses used all 42 matched records; records for control patients matched to more than one intervention patient were retained in full and carry identical baseline values, so no de-duplication was applied. Differing denominators therefore reflect missing data only (Charlson Comorbidity Index available for 38 of 42 records, serum albumin for 35, FEV1 for 41); that the Charlson denominator coincides with the number of unique control individuals (38) is coincidental

*ASA* American Society of Anesthesiologists, *BMI* body mass index, *CCI* Charlson Comorbidity Index, *COPD* chronic obstructive pulmonary disease, *CR2* bias-reduced cluster-robust variance estimator, *CROSS* ChemoRadiotherapy for Oesophageal cancer followed by Surgery, *ECOG* Eastern Cooperative Oncology Group, *FEV1* forced expiratory volume in 1 s, *FLOT* fluorouracil, leucovorin, oxaliplatin and docetaxel, *IQR* interquartile range, *ITT* intention-to-treat cohort, *MC* matched control cohort, *N/A* not applicable (no matched control data available), *PP* per-protocol cohort, *SD* standard deviation

Neoadjuvant therapy was administered to 96.2% (ITT), 95.5% (PP) and 95.2% (MC) of patients, the most frequent form being chemoradiotherapy following the CROSS protocol (73.1% (ITT), 68.2% (PP), 71.4% (MC)), the remaining patients having received perioperative FLOT chemotherapy (23.1% (ITT), 27.3% (PP), 23.8% (MC)) [26–28]. Regarding the type of surgical procedure, the matched controls show a higher percentage of minimally invasive (9.5%) and robotic (26.2%) surgery in comparison to the PP group (4.5 and 9.1%), but also more open procedures (19 vs. 0%). The predominant surgical approach in the PP group was a hybrid procedure (86.4 vs. 45.2% in the MC group) with laparoscopic gastric mobilization and open transthoracic esophagectomy.

We also assessed all enrolled participants by a comprehensive geriatric assessment addressing nutritional status and sarcopenia, dysphagia, cognition, mood, social status, clinical frailty and mobility as listed in the Material and methods section. Equivalent measurements for the matched controls were not available. There were no relevant differences between the ITT and the PP cohort (Table 2). Measures of dysphagia and clinical frailty were most frequently outside the normal range while all other domains ranged well within the age-adjusted expected range. Overall, study participants were mostly non-frail to mildly frail.

**Table 2:** Geriatric and functional assessment of the interventional cohorts

Measure	Statistic	PP (*n* = 22)	ITT (*n* = 26)
Weight loss [n (%)]		*N* = 22	*N* = 26
< 10%		18 (81.8%)	19 (73.1%)
≥ 10%		4 (18.2%)	7 (26.9%)
NRS (0–7)	Median (IQR)	3.0 (2.0–4.0)	3.0 (2.0–4.0)
Ogilvie dysphagia score (0–4) [n (%)]		*N* = 22	*N* = 26
0		14 (63.6%)	16 (61.5%)
1		4 (18.2%)	6 (23.1%)
2		3 (13.6%)	3 (11.5%)
3		1 (4.5%)	1 (3.8%)
Hand grip strength right (kg)	Mean (SD)	32.1 (11.0)	30.8 (11.0)
Hand grip strength left (kg)	Mean (SD)	28.1 (10.8)	27.5 (10.5)
Serum sodium (mmol/L)	Mean (SD)	139.3 (3.2)	138.9 (3.6)
Clinical Frailty Scale — CFS (1–9) [n (%)]		*N* = 22	*N* = 26
2 (well)		6 (27.3%)	6 (23.1%)
3 (managing well)		11 (50.0%)	11 (42.3%)
4 (vulnerable)		4 (18.2%)	8 (30.8%)
6 (moderately frail)		1 (4.5%)	1 (3.8%)
Modified Glasgow Prognostic Score — mGPS (0–2) [n (%)]		*N* = 22	*N* = 25
0		18 (81.8%)	19 (76.0%)
1		2 (9.1%)	4 (16.0%)
2		2 (9.1%)	2 (8.0%)
MMSE (0–30)	Median (IQR)	28.0 (28.0–30.0)	28.0 (27.0–29.2)
Geriatric Depression Scale — GDS (0–15)	Median (IQR)	1.0 (0.0–2.0)	1.0 (0.0–2.0)
Social status — Nikolaus (0–25)	Median (IQR)	21.0 (20.0–23.0)	22.0 (20.0–22.0)
Timed up-and-go — TUG (s)	Median (IQR)	10.0 (9.0–11.0)	10.0 (9.5–12.0)
DASI (0–58.2)	Median (IQR)	44.8 (37.3–54.3)	42.7 (34.1–51.6)
DEMMI (0–100)	Median (IQR)	100.0 (100.0–100.0)	100.0 (100.0–100.0)
GPAQ MET average/day	Median (IQR)	289.3 (137.1–466.1)	289.3 (137.1–453.2)

No equivalent assessment was performed in the matched control cohort. Continuous and ordinal outcomes: mean (SD) for normally distributed variables, median (IQR) for non-normally distributed variables. Categorical outcomes: n (%)

*CFS* Clinical Frailty Scale, *DASI* Duke Activity Status Index, *DEMMI* de Morton Mobility Index, *GDS* Geriatric Depression Scale, *GPAQ* Global Physical Activity Questionnaire, *IQR* interquartile range, *ITT* intention-to-treat cohort, *MET* metabolic equivalent of task, *mGPS* modified Glasgow Prognostic Score, *MMSE* Mini-Mental State Examination, *NRS* nutritional risk screening, *PP* per-protocol cohort, *SD* standard deviation, *TUG* timed up-and-go

### Primary outcome

Retention during the preoperative and in-hospital study period was high, whereas completion of the 90-day outpatient follow-up was lower. All 26 patients completed visits V1 and V2, 24 patients completed visits V3 and V4, while the outpatient follow-up visit was completed by 19 of 26 patients. Reasons for not completing study visits were premature death, dropout after switch to best supportive care, and particularly for V5 loss to follow-up. Overall data completeness was 76.25%. As one parameter of feasibility, Table 3 presents the adherence to the training program as self-reported by the patients at two distinct time points with a two-week interval during the prehabilitation period. At the first instance of inquiry, 16 (61.5%) of the 26 participants reported completely adhering to the training as previously instructed. Six (23.1%) patients mostly and one (3.8%) only rarely followed the program. No data were collected on the remaining three (11.5%) patients. At the second time of inquiry, three (11.5%) participants reported completely, one (3.8%) mostly, three (11.5%) occasionally and one (3.8%) never adhering to the training plan. For most patients (69.2%), however, no data were collected. Applying a worst-case assumption in which all 18 participants with missing data at the second check-in are counted as non-adherent, the complete adherence rate would be 11.5% (3/26) and the complete-or-mostly-complete adherence rate would be 15.4% (4/26).

**Table 3:** Adherence to the training program in the ITT cohort, as self-reported by patients during the intervention period

Execution of training	First inquiry *n*	First inquiry %	Second inquiry *n*	Second inquiry %
Always	16	61.5	3	11.5
Mostly	6	23.1	1	3.8
Occasionally	0	0.0	3	11.5
Partly	0	0.0	0	0.0
Rarely	1	3.8	0	0.0
Never	0	0.0	1	3.8
No data	3	11.5	18	69.2

Percentages refer to all 26 patients of the ITT cohort

*ITT* intention-to-treat cohort

As shown in Table 4, an adjustment of training intensity was necessary for three patients at the first check-in during the program, two (7.7%) needing a reduction of intensity and one (3.8%) an increase. At the second check-in, successful inquiries were only documented for 7 (26.9%) participants. The only adjustment was an increase in training intensity for one (3.8%) patient.

**Table 4:** Necessity for adjustment of training intensity in the ITT cohort, as self-reported by patients during the intervention period

Adjustment in training intensity	First inquiry *n*	First inquiry %	Second inquiry *n*	Second inquiry %
No adjustment needed	19	73.1	6	23.1
Increase of intensity	1	3.8	1	3.8
Decrease of intensity	2	7.7	0	0.0
No data	4	15.4	19	73.1

Percentages refer to all 26 patients of the ITT cohort

*ITT* intention-to-treat cohort

The quota for completion of the assessment tests differs between the type and timing of testing (Online Resource 2). For every test scheduled at both baseline assessment (V2) and after the prehabilitation period (V3), the rate of successful data collection was significantly higher at V2. Here, all but one of the tests were completed for more than 80% of the patients. At V3, this was only achieved for the NRS. BIVA had a notably lower completion rate (57.69% at V2, 38.46% at V3) compared to the other tests. The assessments at hospital discharge (V4) and at the 90-day follow-up (V5) show more successful data collection for Clavien–Dindo score (92.31%) and 90-day mortality (88.46%) than for BMI (76.92% at V4, 65.38% at V5).

### Safety

No intervention-related adverse events were observed during the prehabilitation period. Specifically, no falls, training-related injuries, or cardiovascular events attributable to the prehabilitation program were documented in any participant.

### Secondary outcome

The secondary outcome parameters for the PP and MC groups are demonstrated in Table 5. Exploratory comparisons did not show statistically significant differences in overall postoperative complications (Clavien–Dindo distribution, *p* = 0.138), major complications (Clavien–Dindo ≥ grade 3: 45.5% vs. 61.9%; OR 0.52, 95% CI 0.18–1.45; *p* = 0.213), anastomotic leakage (13.6% vs. 21.4%; OR 0.63, 95% CI 0.14–2.28; *p* = 0.496), hospital mortality (4.5% vs. 2.5%; OR 1.84, 95% CI 0.14–23.69; *p* = 0.610), or postoperative length of stay (13 vs. 15.5 days; *p* = 0.189). ICU readmission was lower in the prehabilitation group compared to matched controls (4.5% vs. 26.2%; OR 0.19, 95% CI 0.02–0.90; *p* = 0.035). In the pre-specified sensitivity analysis adjusting for surgical approach, the difference in ICU readmission was no longer significant (adjusted OR 0.30, 95% CI 0.03–1.63; *p* = 0.172); other outcomes remained non-significant (Online Resource 3).

**Table 5:** Postoperative complications, anastomotic leakage, ICU readmission, hospital mortality and duration of hospital stay in the PP and MC cohorts

Outcome variable	PP (*n* = 22)	MC (*n* = 42)	OR (95% CI)	*p* value PP vs. MC
Clavien–Dindo grade [n (%)]			N/A	0.138
Grade 0	7 (31.8%)	9 (21.4%)		
Grade 1	3 (13.6%)	3 (7.1%)		
Grade 2	2 (9.1%)	4 (9.5%)		
Grade 3a	8 (36.4%)	15 (35.7%)		
Grade 3b	–	5 (11.9%)		
Grade 4a	–	2 (4.8%)		
Grade 4b	1 (4.5%)	3 (7.1%)		
Grade 5	1 (4.5%)	1 (2.4%)		
Clavien–Dindo ≥ grade 3 [n (%)]	10 (45.5%)	26 (61.9%)	0.52 (0.18–1.45)	0.213
Anastomotic leakage [n (%)]	3 (13.6%)	9 (21.4%)	0.63 (0.14–2.28)	0.496
ICU readmission [n (%)]	1 (4.5%)	11 (26.2%)	0.19 (0.02–0.90)	0.035
Hospital mortality [n (%)]	1 (4.5%)	1 (2.5%) (*N* = 40)^a^	1.84 (0.14–23.69)	0.610
Postoperative hospital stay (days) [median (IQR)]	13 (11–20.8)	15.5 (12–23.8)	N/A	0.189

^a^ Missing for one control patient who entered the matched cohort twice under matching with replacement; complete-case analysis therefore removes two of the 42 control records

For binary outcomes (Clavien–Dindo ≥ grade 3, anastomotic leakage, ICU readmission, hospital mortality), OR, 95% CI and p value were all derived from Firth penalized logistic regression (penalized likelihood ratio test), ensuring internal consistency of all three quantities under sparse-data conditions. The Clavien–Dindo distribution was tested with the Wilcoxon rank-sum test (ordinal; does not account for the matched structure). Postoperative hospital stay was tested with cluster-robust linear regression (CR2) on log1p-transformed values and is shown as median (IQR). All matched control duplicates were retained; for the continuous outcome (postoperative hospital stay), clustering by patient ID accounts for the matched-with-replacement design

*CI* confidence interval, *CR2* bias-reduced cluster-robust variance estimator, *ICU* intensive care unit, *IQR* interquartile range, *MC* matched control cohort, *N/A* not applicable, *OR* odds ratio, *PP* per-protocol cohort

### Preoperative functional changes (V2 to V3)

No statistically significant deterioration was observed across functional assessment domains between the baseline (V2) and post-prehabilitation (V3) assessments in the PP cohort, consistent with the primary goal of functional maintenance during neoadjuvant therapy. Full results including standardized effect estimates (SD units), 95% CI, and p values for each assessment domain are reported in Online Resource 4.

## Discussion

This prospective feasibility study demonstrates that a structured, home-based, multimodal prehabilitation program integrated with geriatric co-management can be implemented safely in patients aged ≥ 65 years awaiting esophagectomy for esophageal cancer. COVID-19-related constraints affected the study intervention, which nevertheless remained feasible. The main findings of the study are threefold. First, recruitment and perioperative retention were feasible, with nearly all participants completing the preoperative and in-hospital study visits. Second, early self-reported adherence to the prescribed training program was encouraging, although longitudinal documentation declined markedly toward the end of the intervention period. Third, exploratory comparisons with matched historical controls did not show statistically significant differences in overall postoperative morbidity, anastomotic leakage, hospital mortality, or postoperative length of stay. ICU readmission was lower in the prehabilitation cohort (*p* = 0.035), although this difference did not persist after adjustment for surgical approach and must be interpreted cautiously given the small number of events and the exploratory design.

Feasibility in this setting should be interpreted beyond simple completion of the intervention. In older patients with esophageal cancer, the preoperative window is often constrained by neoadjuvant treatment, nutritional impairment, dysphagia, and anxiety before major surgery. Against this background, the high rate of complete or mostly complete self-reported adherence at the first check-in indicates that a home-based prehabilitation program is acceptable to many patients in this pathway. At the same time, the large proportion of missing adherence data at the second check-in illustrates a central implementation challenge. Unsupervised home-based prehabilitation requires reliable longitudinal monitoring. Future programs should therefore include predefined documentation workflows, standardized telephone assessments, digital adherence tools, wearable activity tracking, or nurse- and physiotherapist-led follow-up contacts to ensure that adherence and training adaptations are captured consistently.

A distinctive feature of the present program was the integration of comprehensive geriatric assessment and geriatric co-management into the preoperative pathway. This approach is particularly relevant because chronological age alone does not adequately reflect perioperative vulnerability. Frailty, mobility limitations, cognitive impairment, depressive symptoms, malnutrition, dysphagia, social support, and polypharmacy may all influence postoperative recovery. In the present cohort, most participants were non-frail to mildly frail, while severe frailty was uncommon. This likely reflects selection within the surgical assessment, as patients with pronounced frailty cannot be treated with major oncological surgery and are directed toward non-surgical treatment strategies. Consequently, the potential effect size of geriatric co-management may have been attenuated in this relatively fit older cohort. Future studies should consider frailty-stratified inclusion criteria or predefined subgroup analyses to identify those patients most likely to benefit from geriatric prehabilitation. Additionally, structured prehabilitation programs may provide a viable intervention for patients under conservative care, aimed at improving baseline fitness to a threshold that permits a reconsideration of eligibility for major oncological surgery.

Exploratory postoperative outcome analyses did not demonstrate statistically significant differences between the prehabilitation cohort and matched historical controls. This finding is not unexpected given the small sample size, the feasibility design, and the limited power to detect differences in postoperative morbidity. Importantly, the matched control cohort showed more favorable ECOG performance status and CCI scores despite matching for age group, ASA class, neoadjuvant therapy, and BMI category. These residual baseline imbalances may have biased comparisons against the intervention group. Baseline forced expiratory volume in 1 s was also higher in the matched controls (2.7 ± 0.7 L vs. 2.4 ± 0.6 L in the per-protocol cohort; *p* = 0.044), although absolute spirometric values were not adjusted for height, age or sex and percent-predicted values were not available. All three imbalances therefore favoured the control cohort, so confounding by measured baseline pulmonary function, performance status or comorbidity burden would be expected to bias the comparison against the prehabilitation group rather than in its favour. In addition, differences in surgical approach between groups may have introduced further confounding (Online Resource 3). ICU readmission was lower in the prehabilitation group (4.5% vs. 26.2%; OR 0.19, 95% CI 0.02–0.90; *p* = 0.035, Firth penalized likelihood ratio test). However, this result must be interpreted with caution. Only one event occurred in the prehabilitation group versus eleven in controls, yielding a wide confidence interval that barely excludes unity. Moreover, in the pre-specified sensitivity analysis adjusting for surgical approach the association was attenuated and no longer statistically significant (adjusted OR 0.30, 95% CI 0.03–1.63; *p* = 0.172), indicating that the unadjusted difference is not robust to differences in operative technique between cohorts. The study was not formally powered for this endpoint, residual baseline imbalances in ECOG and CCI may have biased the comparison, and the finding could reflect unmeasured confounding rather than a causal effect of prehabilitation. Nonetheless, the direction and magnitude of the effect — a clinically plausible reduction in ICU readmission through improved preoperative cardiopulmonary reserve — is consistent with the mechanistic rationale of prehabilitation and warrants replication in an adequately powered randomized trial. This endpoint may be particularly relevant in esophageal surgery, where pulmonary complications and frailty increase the risk of postoperative deterioration.

These findings are consistent with the broader prehabilitation literature, in which improvements in functional capacity or perioperative resilience do not consistently translate into measurable reductions in overall postoperative complication rates. Several factors may explain this discrepancy. First, postoperative morbidity after esophagectomy is multifactorial and influenced by tumor stage, neoadjuvant therapy, nutritional status, pulmonary risk, surgical technique, and institutional perioperative pathways. Second, conventional endpoints such as overall complication rate or Clavien–Dindo classification may be insufficiently sensitive to detect benefits of prehabilitation in small or moderately sized cohorts. Third, clinically meaningful effects may become apparent in more specific endpoints, such as pulmonary complications, failure to rescue, postoperative functional recovery, discharge destination, quality of life, or timely initiation of adjuvant therapy. Future trials in esophageal cancer surgery should therefore combine traditional surgical endpoints with patient-centered and resilience-oriented outcomes.

Several limitations must be acknowledged. First, this was a single-center feasibility study with a small sample size and no formal power calculation for clinical endpoints. Accordingly, all comparisons of postoperative outcomes must be considered exploratory. Second, the use of matched historical controls introduces an inherent risk of residual confounding. Although matching was performed for clinically relevant variables, baseline differences in ECOG performance status, CCI and FEV1 remained, and geriatric assessment data were not available for the control group. Third, heterogeneity in surgical approach may have influenced postoperative outcomes independently of the prehabilitation intervention. Fourth, adherence was self-reported and incompletely documented, particularly toward the end of the program, limiting conclusions regarding dose–response relationships. Fifth, completion rates for several functional, nutritional, and quality-of-life assessments decreased from baseline to the preoperative follow-up visit (V3 completion ranging from 38.5% to 80.8% across instruments; Online Resource 2), and outpatient 90-day follow-up was incomplete. The differential V3 completion rates introduce potential survivorship bias in the V2-to-V3 analysis, as patients successfully completing V3 assessments may be systematically fitter than non-completers; a formal comparison of baseline characteristics between V3-completers and non-completers was not performed and remains an important analysis for future work. Additionally, no pre-specified stop/go feasibility thresholds were defined prior to study initiation; the interpretation of feasibility is therefore based on qualitative judgement and comparison with published benchmarks rather than prospectively defined decision criteria. Finally, the intervention combined exercise training, respiratory training, balance exercises, and geriatric medication review, making it impossible to isolate the contribution of individual components.

Despite these limitations, the present study provides relevant implementation data for future perioperative trials in older patients undergoing esophagectomy. The results suggest that geriatric co-managed prehabilitation is acceptable and safe in this population, but that home-based programs require more robust monitoring to ensure adherence documentation and timely training adaptation. Future multicenter randomized trials should use standardized prehabilitation protocols, frailty-stratified designs, predefined adherence metrics, and endpoints that capture both surgical morbidity and postoperative resilience. However, as exemplified for adjuvant training by the CHALLENGE trial, structured exercise with mandatory in-person support sessions may be needed to demonstrate meaningful improvements [29].

## Conclusion

This prospective feasibility study demonstrates that a structured, home-based, multimodal prehabilitation program integrated with comprehensive geriatric assessment and geriatric co-management is feasible and safe in patients aged ≥ 65 years undergoing esophagectomy for esophageal cancer even despite COVID-19-related constraints. Exploratory comparisons with matched historical controls did not show significant differences in overall postoperative morbidity, anastomotic leakage, hospital mortality, or length of stay. Residual baseline imbalances in ECOG performance status and CCI despite matched-cohort design should be considered when interpreting these null findings. ICU readmission was lower in the prehabilitation cohort (4.5% vs. 26.2%; OR 0.19, 95% CI 0.02–0.90; *p* = 0.035), but not after adjustment for surgical approach (adjusted OR 0.30, 95% CI 0.03–1.63; *p* = 0.172); given the small number of events, the wide confidence interval and this attenuation, the finding should be interpreted as hypothesis-generating and requires replication in an adequately powered randomized trial. Incomplete adherence documentation and reduced follow-up completion highlight key methodological challenges of home-based prehabilitation. Future adequately powered multicenter trials should incorporate standardized, ideally in-house monitoring strategies, frailty-stratified designs, and clinically meaningful perioperative and functional endpoints.

## Supplementary Information

Below is the link to the electronic supplementary material.

Supplementary Material 1 (PDF 850 KB)

## Data Availability

The aggregated datasets analyzed during the current study are available from the corresponding author on reasonable request. To protect participant privacy and comply with data protection regulations, individual participant data will not be made available.

## Code availability

The statistical analyses were performed with custom R scripts. R scripts are available upon request. Hans Schlößer received support for a clinical trial by AstraZeneca and research support by Peptides and Elephants.

## Declarations

### Clinical trial registration

The study was registered at ClinicalTrials.gov under the identifier NCT05167682 on 9 December 2021, after enrolment of the first participant on 18 June 2021, and is therefore retrospectively registered.

### Permissions

No previously published figures, tables, questionnaires or text passages are reproduced in this manuscript. Standardized clinical instruments used in the study (DASI, GPAQ, NRS 2002, Ogilvie dysphagia score, CFS, TUG, DEMMI, MMSE, GDS, SF-36, mGPS) are cited in the Material and methods section but are not reproduced. Instruments were licensed when applicable.
