# Supplementary material for: Geriatric co-managed prehabilitation before esophagectomy in patients aged ≥ 65 years with esophageal cancer: a prospective feasibility study with matched historical controls
Source: Langenbecks Arch Surg. 2026 Sep 29;411(1):253. doi: 10.1007/s00423-026-04285-y (PMC13624024; doi:10.1007/s00423-026-04285-y)
Supplement: Supplementary file 1 [file 423_2026_4285_MOESM1_ESM.pdf]

Online Resource 1 CONSORT flow diagram depicting patient selection and cohort assembly

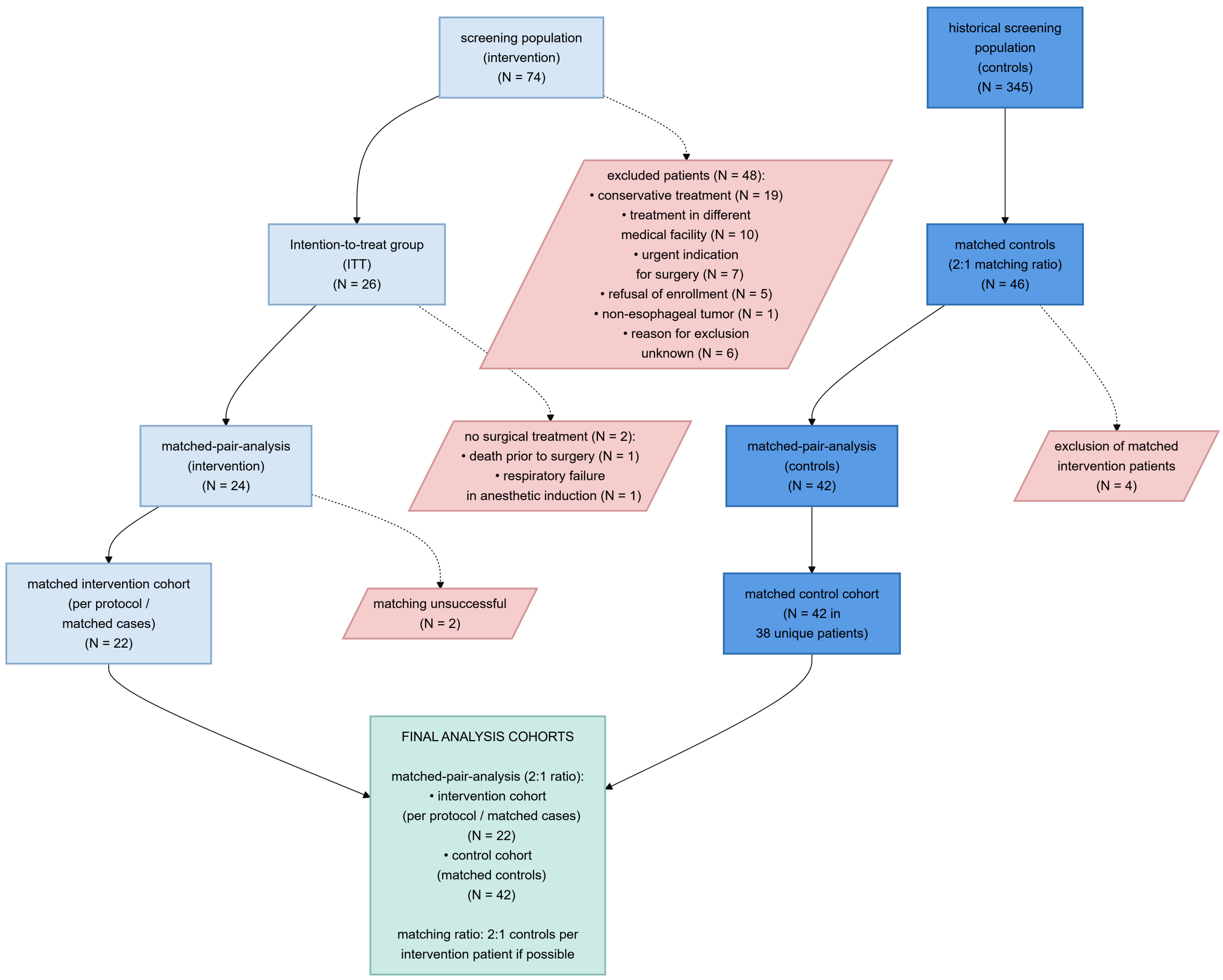

Light blue boxes denote the intervention stream, dark blue boxes the historical control stream, parallelograms patients not entering the next analysis step, and the green box the final analysis cohorts.

ITT intention-to-treat cohort

Online Resource 2 Completion rate of assessment tests in the ITT cohort

Measure	V2 n	V2 %	V3 n	V3 %	V4 n	V4 %	V5 n	V5 %
GPAQ	24	92.31	18	69.23				
NRS	25	96.15	21	80.77				
SF-36	26	100.00	15	57.69				
6-minute walk test	23	88.46	17	65.38				
Pulmonary function / spirometry	23	88.46	14	53.85				
BIVA	15	57.69	10	38.46				
DASI	23	88.46						
DEMMI	24	92.31						
GDS	25	96.15						
MMSE	24	92.31						
Social situation (Nikolaus)	25	96.15						
Hand grip strength	24	92.31						
TUG	23	88.46						
BMI					20	76.92	17	65.38
Clavien–Dindo score					24	92.31		
90-day mortality							23	88.46

Percentages refer to all 26 patients of the ITT cohort. Empty cells indicate that the measure was not scheduled at that visit. V2, baseline; V3, after completion of the prehabilitation period; V4, hospital discharge; V5, 90-day follow-up.

BIVA bioelectrical impedance vector analysis, *BMI* body mass index, *DASI* Duke Activity Status Index, *DEMMI* de Morton Mobility Index, *GDS* Geriatric Depression Scale, *GPAQ* Global Physical Activity Questionnaire, *ITT* intention-to-treat cohort, *MMSE* Mini-Mental State Examination, *NRS* nutritional risk screening, *SF-36* 36-Item Short Form Health Survey, *TUG* timed up-and-go

Online Resource 3 Sensitivity analysis: postoperative outcomes adjusted for surgical approach

Outcome	Adjusted OR (95% CI)	Adjusted p value
Clavien–Dindo ≥ grade 3	0.62 (0.19–1.95)	0.410
Anastomotic leakage	1.17 (0.22–6.24)	0.850
ICU readmission	0.30 (0.03–1.63)	0.172
Hospital mortality	1.07 (0.08–13.52)	0.955
Postoperative hospital stay	N/A	0.662

Surgical approach (open, hybrid, minimally invasive or robotic) was entered as a factor covariate. Binary outcomes: Firth penalized logistic regression (OR, 95% CI and p value from the penalized likelihood ratio test). Postoperative hospital stay: cluster-robust linear regression (CR2) on log1p-transformed values, clustering by patient ID. All matched control records were retained.

CI confidence interval, *CR2* bias-reduced cluster-robust variance estimator, *ICU* intensive care unit, *N/A* not applicable, *OR* odds ratio

Online Resource 4 Changes in clinically relevant measures between the baseline pre-prehabilitation (V2) and post-prehabilitation preoperative (V3) visits in the PP cohort

**Measure**	**n (V2)**	**n (V3)**	**n (complete pairs)**	**Estimate (SD units)**	**95% CI lower**	**95% CI upper**	**SE**	**p value**
6-minute walk test (6MWT)	22	19	17	0.15	−0.07	0.38	0.12	0.202
FEV1	21	13	10	0.24	−0.09	0.57	0.17	0.189
NRS	23	20	20	−0.62	−1.08	−0.16	0.23	0.015*
QoL — physical function	24	14	14	0.17	−0.16	0.50	0.17	0.330
QoL — psychological wellbeing	24	14	14	0.24	0.00	0.47	0.12	0.069
Serum albumin	22	21	20	0.13	−0.28	0.53	0.21	0.543
mGPS	23	24	23	−0.28	−0.83	0.26	0.28	0.321
Body weight	24	20	20	0.02	−0.10	0.14	0.06	0.769
BMI	24	20	20	0.03	−0.11	0.17	0.07	0.687
Phase angle (BIVA)	13	8	7	−0.01	−0.50	0.47	0.25	0.963

* $p<0.05$

Linear mixed-effects model (REML) with a random intercept per participant; missing data were handled under a missing-at-random assumption. Outcome variables were Z-standardized before model fitting, so estimates are expressed in SD units and are comparable across measures.

BIVA bioelectrical impedance vector analysis, *BMI* body mass index, *CI* confidence interval, *FEV1* forced expiratory volume in 1 second, *mGPS* modified Glasgow Prognostic Score, *NRS* nutritional risk screening, *PP* per-protocol cohort, *QoL* quality of life, *REML* restricted maximum likelihood, *SD* standard deviation, *SE* standard error
